# Intracochlear Hemorrhage: A Rare Cause of Sudden Sensorineural Hearing Loss

**DOI:** 10.1155/2021/1072047

**Published:** 2021-11-22

**Authors:** Myriam Jrad, Haifa Zlitni, Miriam Boumediene, Atef Ben Nasr, Meriem Bouzrara

**Affiliations:** ^1^Department of Radiology, Charles Nicolle University Hospital, Tunis, Tunisia; ^2^Department of Radiology, La Rabta University Hospital, Tunis, Tunisia

## Abstract

Inner ear hemorrhage is an extremely rare cause of sudden sensorineural hearing loss with few cases reported in the literature. We report the case of a 30-year-old male who presented with a sudden left ear hearing loss, with no tinnitus nor vertigo. The audiogram revealed a profound left sensorineural hearing loss. An MRI of the brain and internal auditory canal was performed 3 weeks after and revealed an increased signal intensity on T1-weighted (T1W) and T2 fluid-attenuated inversion recovery (FLAIR) images in the left cochlea. No other abnormalities were found, in particular no enhancement after intravenous administration of gadolinium. The CISS 3D sequence showed a signal of discreetly lower intensity in the left cochlea compared to the right one. The diagnosis of intracochlear hemorrhage was made. No improvement of the hearing loss has been noted after medical treatment and hyperbaric oxygen therapy.

## 1. Introduction

Sudden sensorineural hearing loss (SSNHL) affects 15 thousand persons worldwide every year [1]. It is generally unilateral and sudden or rapidly progressive [2]. Its physiopathological characteristics are still subjects to debate. 85% to 90% of cases are classified as idiopathic [2, 3]. Spontaneous improvement is reported in 65% of cases within 2 weeks [1]. Various causes can be identified such as vestibular or intralabyrinthine schwannoma, internal auditory canal metastasis, ruptured dermoid cyst, and labyrinthine hemorrhage [4]. Inner ear hemorrhage is an extremely rare cause of SSNHL with few cases reported in the literature. The diagnosis was difficult before the advent of magnetic resonance imaging (MRI) [2].

The aim of this paper is to report a case of an intracochlear hemorrhage as a potential cause of SSNHL in a healthy 30-year-old patient.

## 2. Case Presentation

**Figure 1:**
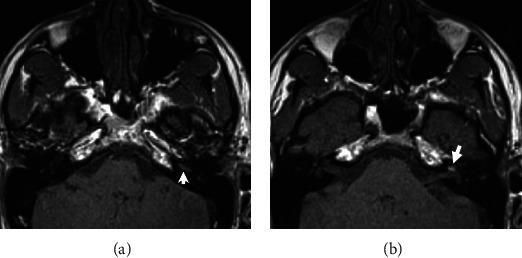
Magnetic resonance imaging of the temporal bone: axial T1-weighted slice before gadolinium injection showing hyperintensity of cochlear basal (a) and second turns (b) of the left ear with a normal signal in the right ear.

**Figure 2:**
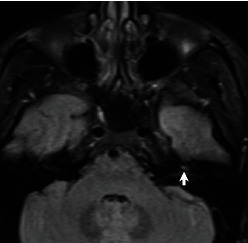
Magnetic resonance imaging: axial T2 FLAIR image showing hyperintensity of the left cochlea.

**Figure 3:**
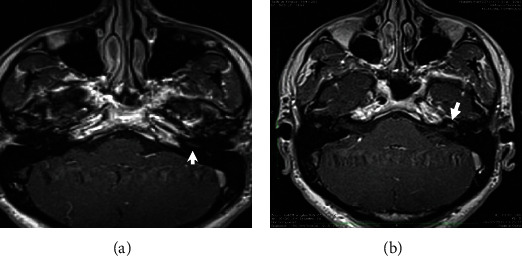
Magnetic resonance imaging of the temporal bone: axial postcontrast T1-weighted slices showing no enhancement after gadolinium injection in the basal (a) and the second (b) turns of the left cochlea.

**Figure 4:**
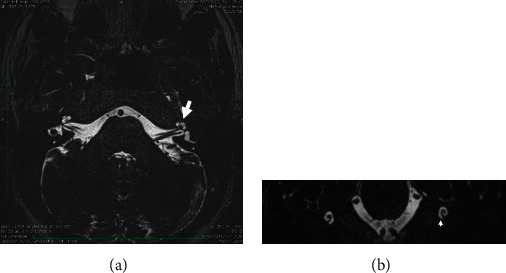
Magnetic resonance imaging of the temporal bone: axial (a) and coronal (b) CISS 3D slices showing a signal of discreetly lower intensity in the left cochlea compared to the right one.

A 30-year-old otherwise healthy male presented with left ear hearing loss that had started 12 hours previously, with no tinnitus nor vertigo. He denied any personal history or family history of otological disease, autoimmune disease, bleeding disorders, or otological or neurologic surgery. The physical examination revealed a lateralized tuning fork test (Weber) to the healthy ear and a normal neurootological examination. The audiogram revealed a profound left SNHL. The patient received corticosteroid, peripheral vasodilator, and antiviral treatment orally for 10 days. Hyperbaric oxygen therapy was started 24 hours after, with a total of 25 meetings. The patient was followed up with physical examination and audiometry 1 day/2. An MRI of the brain and internal auditory canal was performed 3 weeks after and revealed an increased signal intensity on T1-weighted (T1W) (Figure 1) and T2 fluid-attenuated inversion recovery (FLAIR) (Figure 2) images in the left cochlea. No other abnormalities were found, in particular no enhancement after intravenous administration of gadolinium (Figure 3). The CISS 3D sequence showed a signal of discreetly lower intensity in the left cochlea compared to the right one (Figure 4). The diagnosis of intracochlear hemorrhage was made. The patient was addressed to internal medicine and hematology departments where hematological and immunological analyses had been performed with normal results. At the latest audiogram carried out 2 months after, the patient's hearing loss remained profound.

## 3. Discussion

Sudden sensorineural hearing loss (SSHL) is defined as >30 dB hearing loss in at least three contiguous frequencies occurring in a period of less than 72 hours [3]. Physiopathological characteristics are still unclear. Viral infection, microcirculatory disturbance of the labyrinthine, immune factors, and membrane rupture have been proposed as possible causes [5, 6]. However, it remains idiopathic in the majority of cases [2, 5].

Vascular etiology is rare and can be obstructive or hemorrhagic [2, 7]. It affects patients under anticoagulation therapy or suffering from hematological disease [2]. Salome et al. reported that intralabyrinthine bleeding affected most commonly the basal gyrus of the cochlea and the vestibule close to the oval window [2]. Improvements in magnetic resonance imaging allow for better characterization of more subtle inner ear pathologies [8]. The American Academy of Otolaryngology-Head and Neck Surgery (AAO-HNS) Foundation recommends audiometry and MRI scans of the middle and inner ear in cases of SSHL [3].

Fitzgerald and Mark found that MRI of the temporal bone, cerebellopontine angle, and brain showed abnormal results in 24 of 78 patients (31%) with SSNHL [9]. Wu et al. in their series of 112 patients with SSHL found abnormal magnetic resonance images in 13 (11.6%) cases, retrocochlear pathology in 6 cases, and inner ear hemorrhage in 7 cases [5].

In our case, we found a high signal on T1-weighted and 3D FLAIR images without enhancement after gadolinium injection.

The perilymph and the endolymph are isointense compared to the cerebrospinal fluid. A hyperintense T1 signal can be found in cases of high protein concentration and decreased blood flow and in the presence of fat or methaemoglobin [2, 4]. Fat-suppressed T1-weighted images can be used to differentiate fat from blood; however, lipomas are extremely rare in this location [5, 6].

A hyperintense signal in 3D FLAIR images is the expression of a modification in the inner ear protein composition. It can be found in the presence of an acute inflammatory process or hemorrhage [4]. In our case, the MRI was performed after 3 weeks, so the hypothesis of acute inflammation can be dismissed. The presence of a hyperintense signal in both T1 W1 and 3D FLAIR images with or without contrast enhancement is consistent with the diagnosis of intracochlear hemorrhage. FLAIR sequence is a T2-weighted sequence with inversion of the signal of CSF, endolymph and perilymph. Pathologies of the inner ear including increased protein concentration (labyrinthitis) or intralabyrinthine methaemoglobin affect the relaxation time of fluid and appear hyperintense.

**Figure 5:**
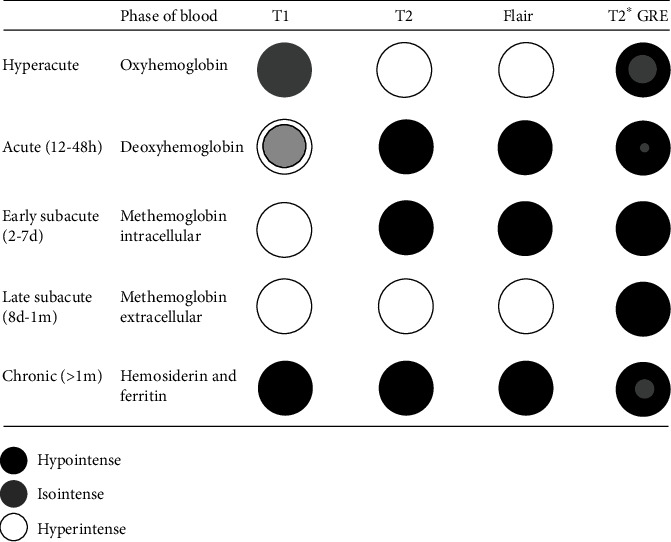
The evolution over time of the MRI haemoglobin aspect in hemorrhage.

The evolution of haemoglobin in hemorrhage passes through predictable stages from oxyhaemoglobin (<24 h), desoxyhaemoglobin (1-3 days), intracellular methaemoglobin (3-7 days), extracellular methaemoglobin (3-7 days), and finally hemosiderin (14 days) (Figure 5). In the subacute stage, the presence of methaemoglobin in the labyrinth will produce high signal intensity on both T1 and FLAIR images. However, haemosiderin appears hypointense intensity on T1 and FLAIR. In order to increase the likelihood of detecting possible intralabyrinthine hemorrhage, MRI should be performed in the subacute stage, 3-14 days after symptom onset.

The treatment of SSHL, even when the etiology is found, remains subject to debate. The prognosis in cases of inner ear hemorrhage is poor. In Wu et al.'s series, none of the seven patients showed an improvement in hearing loss [5]. We noted the same evolution in our case. Initial and final hearing levels, in Ryu et al.'s series of 12 patients, were 98.0 ± 31.1 dB and 87.7 ± 33.1 dB. He concluded that high signals on 3D FLAIR images correlate with vestibular dysfunction and poor hearing prognosis in patients presenting SSHL [10].

The presence of inner ear hemorrhage, especially in an otherwise healthy patient, can be the expression of aplastic sickle cell disease, leukemia, systemic lupus erythematosus, endolymphatic sac tumors, or Von Hippel-Lindau syndrome [2, 8]. In our case, no abnormality was found after hematological and immunological explorations.

## 4. Conclusion

MRI is considered a sensitive imaging method to identify lesions in the cerebellopontine angle, internal auditory canal, and inner ear. It must be performed in patients with SSNHL. Various causes can be diagnosed such as multiple sclerosis, cerebellopontine angle or internal auditory canal tumors, labyrinthitis, stroke, or hemorrhage. Although rare, inner ear hemorrhage should be evocated. This minor hemorrhage may be the first complication of anticoagulation therapy and hematological or autoimmune disease.

## Data Availability

No data were used to support this study.
